# Smartphone-Based Survey and Message Compliance in Adults Initially Unready to Quit Smoking: Secondary Analysis of a Randomized Controlled Trial

**DOI:** 10.2196/56003

**Published:** 2024-06-07

**Authors:** Clayton Ulm, Sixia Chen, Brianna Fleshman, Lizbeth Benson, Darla E Kendzor, Summer Frank-Pearce, Jordan M Neil, Damon Vidrine, Irene De La Torre, Michael S Businelle

**Affiliations:** 1 Department of Health Behavior Gillings School of Global Public Health University of North Carolina at Chapel Hill Chapel Hill, NC United States; 2 TSET Health Promotion Research Center Stephenson Cancer Center University of Oklahoma Health Sciences Center Oklahoma City, OK United States; 3 Department of Biostatistics and Epidemiology Hudson College of Public Health The University of Oklahoma Health Sciences Center Oklahoma City, OK United States; 4 Department of Family and Preventive Medicine University of Oklahoma Health Sciences Center Oklahoma City, OK United States; 5 Department of Health Outcomes and Behavior Moffitt Cancer Center Tampa, FL United States

**Keywords:** just-in-time adaptive intervention, tailored messaging, smoking cessation, mobile health, survey compliance, phase-based model, smoking, smoker, survey, smokers, messaging, smartphone, efficacy, pilot randomized controlled trial, adult smokers, linear regression, age, intervention engagement, engagement

## Abstract

**Background:**

Efficacy of smartphone-based interventions depends on intervention content quality and level of exposure to that content. Smartphone-based survey completion rates tend to decline over time; however, few studies have identified variables that predict this decline over longer-term interventions (eg, 26 weeks).

**Objective:**

This study aims to identify predictors of survey completion and message viewing over time within a 26-week smoking cessation trial.

**Methods:**

This study examined data from a 3-group pilot randomized controlled trial of adults who smoke (N=152) and were not ready to quit smoking within the next 30 days. For 182 days, two intervention groups received smartphone-based morning and evening messages based on current readiness to quit smoking. The control group received 2 daily messages unrelated to smoking. All participants were prompted to complete 26 weekly smartphone-based surveys that assessed smoking behavior, quit attempts, and readiness to quit. Compliance was operationalized as percentages of weekly surveys completed and daily messages viewed. Linear regression and mixed-effects models were used to identify predictors (eg, intervention group, age, and sex) of weekly survey completion and daily message viewing and decline in compliance over time.

**Results:**

The sample (mean age 50, SD 12.5, range 19-75 years; mean years of education 13.3, SD 1.6, range 10-20 years) was 67.8% (n=103) female, 74.3% (n=113) White, 77% (n=117) urban, and 52.6% (n=80) unemployed, and 61.2% (n=93) had mental health diagnoses. On average, participants completed 18.3 (71.8%) out of 25.5 prompted weekly surveys and viewed 207.3 (60.6%) out of 345.1 presented messages (31,503/52,460 total). Age was positively associated with overall weekly survey completion (*P*=.003) and daily message viewing (*P*=.02). Mixed-effects models indicated a decline in survey completion from 77% (114/148) in the first week of the intervention to 56% (84/150) in the last week of the intervention (*P*<.001), which was significantly moderated by age, sex, ethnicity, municipality (ie, rural/urban), and employment status. Similarly, message viewing declined from 72.3% (1533/2120) in the first week of the intervention to 44.6% (868/1946) in the last week of the intervention (*P*<.001). This decline in message viewing was significantly moderated by age, sex, municipality, employment status, and education.

**Conclusions:**

This study demonstrated the feasibility of a 26-week smartphone-based smoking cessation intervention. Study results identified subgroups that displayed accelerated rates in the decline of survey completion and message viewing. Future research should identify ways to maintain high levels of interaction with mobile health interventions that span long intervention periods, especially among subgroups that have demonstrated declining rates of intervention engagement over time.

**Trial Registration:**

ClinicalTrials.gov NCT03405129; https://clinicaltrials.gov/ct2/show/NCT03405129

## Introduction

### Overview

The phase-based model (PBM) argues that smoking cessation intervention messages should be customized to each phase of readiness to quit smoking (ie, motivation, precessation, cessation, and maintenance), thereby meeting the individual where they are and providing targeted and relevant intervention programming [1]. Application of dynamic models, like the PBM, to mobile health (mHealth) interventions for smoking cessation may be especially relevant since 80% of smokers are not ready to quit smoking within the next 30 days [2]. Yet, people who smoke may rapidly cycle through decisions to initiate, continue, and end smoking cessation attempts [2]. Smartphone-based surveys are increasingly being used to identify predictors of health behaviors (eg, smoking and alcohol use) [3-5] and inform mHealth intervention messaging via applied theoretical frameworks like PBM [6-9].

The efficacy of smartphone-based interventions depends not only on adequate intervention content but also on the level and duration of exposure to that content. For instance, SMS text messaging–based smoking cessation interventions have demonstrated efficacy in increasing quit rates, with one meta-analysis showing a 36% higher quit rate compared with control groups [10]. In addition, a systematic review found that SMS text messaging–based support significantly increased biochemically verified continuous abstinence and 7-day point prevalence abstinence rates [11]. Duration of exposure to intervention content matters. For example, Bricker et al [12] found that the longer individuals used a smoking cessation app, the higher the odds of 12-month abstinence. Notably, compared to those who used the app for only 1 week, those who used the app for at least 4 weeks had 50% higher odds of cessation, and those who used the app for 26 weeks had 397% higher odds of cessation [12]. Despite the demonstrated efficacy of these interventions, there is limited research directly examining predictors of compliance with survey completion and viewing of app-based intervention messages in longer-term smoking cessation interventions.

### Predictors of Survey Compliance

Prior research has identified a general trend of declining mHealth intervention engagement over time. For instance, as study durations surpass 7 days, smartphone-based survey completion rates tend to decline [13-15]. Ottenstein and Werner’s [15] meta-analysis of 488 smartphone survey studies demonstrated lower overall survey compliance with longer study periods and higher total surveys prompted. However, the average duration of these studies was only 16.5 days with the median being only 8 days [15]. Less is known about the extent to which longer study periods (≥1 month) relate to declines in interactions with mHealth apps, especially for interventions that last 6 months or longer. Some studies conducted over shorter periods have demonstrated increased compliance with smartphone-based surveys among women versus men [14,16], older adults versus younger adults [13,14,17], and those without versus those with mental health diagnoses [14,18,19]. Yet, longer studies (≥1 month) have identified fewer differences in survey compliance across participant characteristics [18,20,21]. Other potential predictors of smartphone-based survey compliance include race or ethnicity [22-24], education [23-25], and employment status [26]. Municipality (ie, urban or rural), to our knowledge, has not been used to predict smartphone-based survey compliance. Additional research is needed to identify factors that predict smartphone-based survey completion and message viewing for extended study periods (eg, 26 weeks) and how participant characteristics impact the number of interactions with mHealth interventions over time.

### Purpose of This Study

The purpose of this study was to identify correlates of weekly smartphone-based survey completion and viewing of twice daily prompted messages. This aim is relevant since messages, which were tailored to current readiness to quit smoking, were given to a sample of people who were initially not ready to quit smoking in the next 30 days. Based on previous research, we hypothesized that aggregate survey completion and message viewing would be higher in women, nonminoritized adults, those without a history of a mental health diagnosis, and older adults. We also hypothesized that message compliance would be higher in the intervention versus control groups. Finally, we explored whether participant characteristics were related to the rate of reductions in weekly survey completion and message viewing over the 26-week study period. Ultimately, these findings may be used to improve the overall efficacy of smartphone-based interventions.

## Methods

### Participants and Procedure

This study is a secondary analysis of data collected for a randomized controlled trial of 152 adults who were initially not ready to quit smoking cigarettes in the next 30 days. Eligibility requirements included (1) being at 18 years or older, (2) demonstrating >6th grade English literacy level [27], (3) owning an active Android smartphone with an operating system version of 5.2 or higher and a data plan, (4) agreeing to download the Insight study smartphone app (University of Oklahoma Health Sciences Center) onto their phone, (5) agreeing to complete 26 weekly assessments using the study smartphone app, (6) scoring >1 and <7 on the Readiness to Quit Ladder (ie, no plans to stop smoking within 30 days) [28], (7) proving smoking status by either having an expired carbon monoxide level >7 parts per million (in-person baselines) or texting a picture of their pack of cigarettes to study staff immediately when requested during the enrollment phone call, (8) smoking ≥5 cigarettes every day, and (9) having no contraindications to over the counter nicotine replacement therapy (NRT).

Participants were randomized into one of 3 groups: Phoenix only, Phoenix+NRT, or Factoid. They were prompted to complete 1 weekly smartphone-based survey on Saturday mornings that included questions focused on smoking, other health behaviors, and readiness to quit smoking. The app had 4 distinct study phases and participants could move between phases at will. Participants started the study in the motivation phase and remained in that phase until they used the app to set a smoking quit date. When or if a quit date was set, the app moved to the precessation phase and all participants gained access to smoking cessation resources. Participants automatically moved to the cessation phase when the selected quit date arrived and moved to the maintenance phase when desired. Intervention content and resources were specific based on group assignments. All participants received daily messages. Examples of messages are provided in Table S1 in Multimedia Appendix 1. To alert participants of a new message, smartphones rang and vibrated. In addition, a pop-up box appeared on the smartphone home screen asking participants if they would like to review the study message. If participants clicked “yes,” the app automatically opened and displayed the message. If participants ignored the message or clicked “no,” the message was recorded as unread. A total of 2 daily messages (ie, one 30 minutes after waking and one 60 minutes before bedtime) were prompted during the motivation, precessation, and maintenance phases, and up to 5 messages were prompted per day during the cessation phase (ie, messages were presented at the end of cessation phase surveys). Participants could remain in the intensive cessation phase as long as desired. Those assigned to the Phoenix or Phoenix+NRT groups received messages that were tailored to their current cessation phase. Factoid participants received factual or trivia messages that were not related to smoking cessation across all study phases. Days spent in the cessation phase were excluded from the current analyses.

### Ethical Considerations

All study procedures were approved by the University of Oklahoma Health Sciences Center’s institutional review board (IRB#8814). Written informed consent was obtained either in-person or electronically via REDCap (Vanderbilt University) before study enrollment. Each participant was assigned a unique ID number that was linked to their data. Personally identifying information was stored on encrypted servers and accessible only to research personnel. Written signatures were kept in locked and secure filing cabinets behind a locked door. Participants consented to secondary analysis of data collected in this study. Participants were paid US $30 for completing the baseline survey, US $25 for completing the 26-week follow-up, and those who were abstinent at 25 weeks could earn an additional US $20 for providing a carbon monoxide sample. Study participants were compensated with US $5 for each weekly survey they completed throughout the 26-week study. Up to US $185-$205 could be earned by each participant. All payments were made via Greenphire gift cards.

### Measures

#### Baseline Measures

The baseline survey assessed demographic characteristics including age (years), biological sex (male=1, female=0), education (no formal schooling=0, postgraduate degree=20), employment status (employed=1, unemployed=0), race or ethnicity (non-Hispanic White=1, minoritized race=0), and zip codes used to indicate municipality (rural-urban commuting area codes 4-10=rural, rural-urban commuting area codes 1-3=urban) [29]. All participants were asked if they were ever diagnosed with major depressive disorder, bipolar disorder, schizophrenia or schizoaffective disorder, posttraumatic stress disorder, or any other anxiety disorder. A mental health disorder history variable was created whereby those that endorsed a history of any previous mental health diagnosis were coded as 1 and those without such diagnoses were coded as 0.

#### Weekly Survey Measures

The weekly survey consisted of 32 questions, which covered topics such as daily cigarette consumption, quit attempts, readiness to quit smoking, use of smoking cessation resources, lifestyle factors like exercise, diet, and sleep, and personal impacts of the COVID-19 pandemic.

### Aggregating Survey Completion and Message Viewing

Aggregated person-level survey completion rates were calculated using binary variables for each survey or week (range 1-26 surveys). Incomplete surveys were coded as 0. Second, we calculated aggregated person-level message viewing rates across all study days for the morning (range 1-182), evening (range 1-182), and total messages viewed (range 1-364). Messages that were not opened were coded as 0.

### Analytic Plan

Descriptive statistics, including means and standard deviations, were calculated for continuous variables, and frequency and percentages were calculated for categorical variables. For each predictor, we fit 4 models. First, we used linear regression to examine whether each predictor was associated with person-level aggregate weekly survey compliance. Second, we used linear regression to examine whether each predictor was associated with aggregate morning, evening, and total message viewing compliance. Third, we used generalized linear mixed-effects models to examine whether any of the demographic, mental health, or study group variables moderated the association between weeks in the study (ie, up to 26) and survey completion (binary variable). Fourth, we used linear mixed-effects models to examine whether any of the demographic, mental health, or study group variables moderated the association between weeks in the study (ie, up to 26) and weekly aggregated message viewing (ie, up to 14 messages per week). For both mixed-effects models (survey completion and message viewing), if significant interactions arose, we modeled those associations separately for each predictor level (eg, male, female). Survey and message compliance data collected during the cessation phase were excluded from analyses because participants who reached the intensive cessation phase (ie, 5 messages per day) could end it at any time. All analyses were performed using SAS (version 9.4; SAS Institute Inc).

## Results

### Participant Characteristics

On average, participants were aged 50 (SD 12.5, range 19-75) years and had 13.3 (SD 1.6, range 10-20) years of education. Most participants were female (103/152, 67.8%), identified as White (113/152, 74.3%), and lived in urban areas (117/152, 77%). Over half of the participants were unemployed (80/152, 52.6%) and reported having a history of mental illness (93/152, 61.2%). Participants completed 71.8% (2782/3872) of the prompted weekly surveys and viewed 60.6% of the prompted daily messages (31,503/52,460 total). Table 1 provides descriptive statistics for compliance rates and the demographic and mental health diagnoses relevant to this study. On average, 25.5 (SD 1.8) out of the 26 scheduled weekly surveys during the study period were included in the analyses since they were successfully prompted through the Insight app. Of these included weekly surveys, an average of 18.30 (SD 6.8) surveys were completed by participants. When morning and evening message compliance were aggregated, an average of 345.1 (SD 57.5) messages were presented across the 26 study weeks, of which an average total of 207.3 (SD 96.2) messages were viewed per participant.

Weekly survey completion and daily message viewing decreased across the 26-week study period (all *P* values<.001). For the first week of the study, participants completed 77% (114/148) of prompted surveys and viewed 72.3% (1533/2120) of total messages. In the 26th week of the study, survey completion declined to 56% (84/150), and total messages viewed declined to 44.6% (868/1946). The rate of this decline was 1.1% for morning, evening, and total messages viewed per week, which equates to a 27.7% decrease from the start to the end of the 26-week study. In the following sections, we present associations between each predictor variable and (1) person-level aggregate survey compliance; (2) change in weekly survey completion across the 26-week study period; (3) person-level aggregate morning, evening, and total message compliance; and (4) change in weekly compliance for morning, evening, and total messages viewed across the study period.

**Table 1:** Participant characteristics and weekly survey and message compliance.

Variable		Value (n=152)
Age (years), mean (SD)		50.0 (12.5)
Female, n (%)		103 (67.8)
**Race, n (%)**		
	American Indian	20 (13.1)
	Black	13 (8.6)
	White	113 (74.3)
	Other	6 (4.0)
Urban, n (%)		117 (77.0)
Employed, n (%)		72 (47.4)
Education in years, mean (SD)		13.3 (1.6)
Any mental health diagnosis^a^, n (%)		93 (61.2)
**Compliance^b^, n (%)**		
	Weekly survey	18.3 (71.8)
	Morning messages	103.0 (60.4)
	Evening messages	104.3 (60.9)
	Total messages	207.3 (60.6)
**Study group, n (%)**		
	Phoenix	51 (33.6)
	Phoenix+NRT^c^	50 (32.9)
	Factoid	51 (33.6)

^a^Any mental health diagnosis indicates a diagnosis of depression, anxiety, posttraumatic stress disorder, other anxiety disorder, bipolar disorder, or schizophrenia.

^b^Compliance: n=average messages viewed or weekly surveys completed, %=average percentage completed out of prompted.

^c^NRT: nicotine replacement therapy.

### Predictors and Moderators of Aggregated Survey Compliance

Age was significantly associated with the aggregated weekly survey completion rate (*P*=.003), with an expected 0.5% greater survey compliance for each year increase in age. Notably, age, sex, ethnicity, employment status, and municipality moderated the association between weeks in the study and weekly survey completion. Slower declines were observed in older adults, women, Hispanic/Latino participants, those with rural residence, and those unemployed. Predictors of weekly survey compliance across time are presented in Table 2.

**Table 2:** Moderators of the decline in survey completion during the 26-week smartphone-based smoking cessation intervention.

Variable and modalities					Weekly survey		
					*β^a^*	*P* value^b^	
**Study group**							.16
	Phoenix				–0.024		
	Phoenix+NRT^c^				–0.044		
	Factoid				–0.038		
**Sociodemographic**							
	Age				0.002	<.001	
	**Sex**						<.001
			Female	–0.019			
			Male	–0.068			
	**Race**						.23
			White	–0.038			
			Other	–0.027			
	**Ethnicity**						.02
			Hispanic/Latino	–0.033			
			Non-Hispanic/Latino	–0.082			
	**Municipality**						.03
			Rural	–0.019			
			Urban	–0.040			
	**Employment**						.02
			Employed	–0.044			
			Unemployed	–0.026			
	Education				0.003	.20	
**Any mental health diagnosis^d^**							.81
		Yes			–0.036		
		No			–0.034		

^a^*β* represents the regression slope of the weekly completion rate versus weeks in the study. Estimates for age and years of education are used for interaction terms.

^b^*P* values are for testing the significance of interaction terms.

^c^NRT: nicotine replacement therapy.

^d^Any mental health diagnosis indicates a diagnosis of depression, anxiety, posttraumatic stress disorder, other anxiety disorder, bipolar disorder, or schizophrenia.

### Predictors and Moderators of Aggregate Message Viewing

Age was positively associated with aggregate viewing of morning (*β*=0.005, *P*=.007) and total (*β*=0.004, *P*=.02), but not evening, messages. Age, sex, municipality, employment status, and education significantly moderated the association between weeks in the study and morning and total messages viewed. Slower declines in morning and total message viewing were observed for older adults, female participants, rural residents, and among the unemployed and higher educated. For evening message viewing compliance, only age, sex, and education moderated this relationship. Significant moderators of the decline in message viewing are shown in Table 3.

**Table 3:** Moderators of the decline in message viewing during the 26-week smartphone-based smoking cessation intervention.

Variable and modalities					Morning message					Evening message					Total message		
					*β^a^*		*P* value^b^			*β*		*P* value			*β*		*P* value
**Study group**							.18					.55					.37
	Phoenix			–0.012					–0.011					–0.011			
	Phoenix+NRT^c^			–0.012					–0.012					–0.012			
	Factoid			–0.010					–0.011					–0.010			
**Sociodemographic**																	
	Age			<.001			.18		<.001		<.001			<.001			<.001
	**Sex**						<.001				<.001						<.001
		Female	–0.008					–0.008					–0.008				
		Male	–0.018					–0.017					–0.018				
	**Race/** **Ethnicity**						.77				.45						.55
		White	–0.011					–0.011					–0.011				
		Other	–0.011					–0.010					–0.011				
	**Municipality**						<.001				.08						.002
		Rural	–0.008					–0.009					–0.009				
		Urban	–0.012					–0.012					–0.012				
	**Employment**						.005				.18						.03
		Employed	–0.013					–0.012					–0.012				
		Unemployed	–0.010					–0.010					–0.010				
	Education			0.001			.002		0.002		<.001			0.001			<.001
**Any mental health diagnosis^d^**							.21					.009					.04
	Yes			–0.011					–0.010					–0.010			
	No			–0.012					–0.013					–0.012			

^a^*β* represents the regression slope of the weekly completion rate versus weeks in the study. Estimates for age and years of education are used for interaction terms.

^b^*P* values are for testing the significance of interaction terms.

^c^NRT: nicotine replacement therapy.

^d^Any mental health diagnosis indicates a diagnosis of depression, anxiety, posttraumatic stress disorder, other anxiety disorder, bipolar disorder, or schizophrenia.

## Discussion

### Principal Findings

This study contributes to a growing body of literature that aims to identify predictors of smartphone-based survey completion and interaction with or viewing of mHealth intervention messages over long study periods. Study findings indicated that 71.8% (2782/3872) of weekly surveys were completed and 60.6% (31,503/52,460) of daily messages were viewed by participants over 26 weeks. Results showed that weekly survey compliance and message viewing declined over time, and several variables moderated the rate of decline in weekly survey compliance (ie, age, sex, ethnicity, municipality, employment status) and message viewing (ie, age, sex, municipality, employment status, and years of education). Numerous prior studies have examined predictors of compliance to smartphone-based surveys [3,13,14,18,20,21], and some have identified variables related to compliance over time [30,31]. However, this study is the first to simultaneously assess predictors of both weekly survey completion and viewing of prompted daily messages, and the first to do so over such a long study period (ie, 26 weeks). Findings from this study provide insights into demographic subgroups that should be monitored and addressed to maintain high engagement with mHealth interventions focused on prompting and supporting health behavior change.

### Implications of Variables Related to Weekly Survey Completion

Age was the only measured variable that was significantly related to person-level aggregate survey completion. Our findings are consistent with Comulada et al [17], which examined adherence to self-monitoring of healthy lifestyle behaviors through smartphone-based surveys and photographic food records over 26 weeks. Comulada et al [17] found that age, but not other demographic characteristics, was significantly related to overall survey completion. Furthermore, this study is consistent with other smartphone-based studies with durations ≥1 month that failed to detect differences in compliance across non–age-related demographic variables [18,20,21]. Although aggregate survey completion did not vary across predictor variables (except for age), several variables moderated the rate of decline in weekly survey completion over the 6-month study period. Specifically, slower declines in survey compliance were observed for female versus male participants, non-Hispanic versus Hispanic participants, older versus younger participants, unemployed versus employed participants, and rural versus urban residents. These findings indicate that multiple demographic factors may play important roles in the reduction of engagement with weekly surveys.

### Implications of Variables Related to the Decline in Message Viewing

Similar to survey completion, findings indicated that age was the sole predictor of person-level aggregate message viewing over the 26-week study period. Furthermore, the rate of decline in daily message viewing varied across several demographic factors. Specifically, the reduction in message viewing over 26 weeks was less rapid for female versus male participants (ie, morning and evening messages), those with rural versus urban residency (ie, morning messages), unemployed versus employed individuals (ie, morning messages), and those with more years of education (ie, morning and evening messages). Engagement with intervention content may be tied to intervention efficacy [12,32]. Therefore, targeted support and strategies that aim to boost engagement with smartphone-based interventions may be needed for individuals in subgroups that displayed a greater rate of decline in message viewing, especially for morning messages.

### Strengths and Weaknesses

This study has multiple strengths. While the majority of previous smartphone-based survey studies have examined relatively brief study periods (eg, 7-30 days [13,14,18,20,21]), our findings are among the first to assess smartphone-based survey completion rates and message viewing over a 26-week study period. Despite the extended duration of this study, participants completed 71.8% (2782/3872) of prompted weekly surveys and viewed 60.6% (31,503/52,460) of prompted twice daily messages. Notably, message viewing was not incentivized, which may suggest that participants found the message content engaging. These findings suggest that people who smoke and are not initially ready to quit may engage with a low-burden smartphone app for relatively long periods.

This study also has some notable weaknesses. First, participants were only prompted to complete 1 survey each week. This study design decision was purposefully made to determine the feasibility of this type of low-burden, long-duration mHealth intervention that aimed to motivate and support behavior change in adults who were not initially ready to quit smoking. Thus, this study does not necessarily offer insights about whether more intensive smartphone survey protocols (ie, multiple surveys per day) are acceptable for long-duration mHealth interventions. Second, weekly survey completion was incentivized (ie, US $5 per weekly survey completed). Future research should investigate if observed patterns emerge in real-world nonincentivized settings. Third, Phoenix participants who never set a quit date could have received duplicate messages after 76 days, whereas those who set quit dates were less likely to receive duplicate messages. Factoid participants did not receive duplicate messages. Receiving duplicate messages could have reduced message viewing as participants may not have liked repeated messages. Fourth, the sample was relatively small and was mostly middle-aged, White female individuals from urban areas in Oklahoma and Texas. These findings may not generalize to other groups of people who smoke and those who are ready to quit smoking.

### Conclusions and Future Directions

Overall, this 26-week smoking cessation induction trial showed robust participant engagement with the intervention and weekly prompted surveys and revealed that only age directly influenced overall survey and message engagement over the extended study period. Further, we identified several demographic variables that significantly moderated the decline in weekly survey completion and message viewing, offering insights that could be useful for future personalized intervention development. Future research should determine if these findings replicate in larger trials and whether efforts to increase engagement with smartphone-based health behavior change interventions are fruitful over time. Future interventions could include additional prompts and reminders to complete weekly surveys and read intervention messages for certain subgroups. Further, researchers should investigate if those who are more compliant are more likely to quit smoking by examining how survey completion and message viewing are associated with intervention effectiveness.

## Appendix

Multimedia Appendix 1 Example messages sent to participants (N=152) who are initially not ready to quit smoking enrolled in a smartphone-based smoking cessation trial.

Multimedia Appendix 2 CONSORT-eHEALTH checklist (V 1.6.1).

## Abbreviations

mHealth: mobile health
NRT: nicotine replacement therapy
PBM: phase-based model

## Data Availability

The data sets generated or analyzed during this study are available from the last author upon reasonable request.

## References

[1] Baker TB, Mermelstein R, Collins LM, Piper ME, Jorenby DE, Smith SS, Christiansen BA, Schlam TR, Cook JW, Fiore MC (2011). New methods for tobacco dependence treatment research. Ann Behav Med.

[2] Harris KJ, Bradley-Ewing A, Goggin K, Richter KP, Patten C, Williams K, Lee HS, Staggs VS, Catley D (2016). Recruiting unmotivated smokers into a smoking induction trial. Health Educ Res.

[3] Shiffman S (2009). Ecological momentary assessment (EMA) in studies of substance use. Psychol Assess.

[4] Walters ST, Businelle MS, Suchting R, Li X, Hébert ET, Mun EY (2021). Using machine learning to identify predictors of imminent drinking and create tailored messages for at-risk drinkers experiencing homelessness. J Subst Abuse Treat.

[5] Mun EY, Li X, Businelle MS, Hébert ET, Tan Z, Barnett NP, Walters ST (2021). Ecological momentary assessment of alcohol consumption and its concordance with transdermal alcohol detection and timeline follow-back self-report among adults experiencing homelessness. Alcohol Clin Exp Res.

[6] Bui TC, Sopheab H, Businelle MS, Chhea C, Ly SP, Vidrine JI, Thol D, Frank-Pearce S, Vidrine DJ (2022). Mobile-health intervention for smoking cessation among Cambodian people living with HIV: a mixed-methods pilot study. AIDS Care.

[7] Garey L, Hébert ET, Mayorga NA, Chavez J, Shepherd JM, Businelle MS, Zvolensky MJ (2022). Evaluating the feasibility and acceptability of a mobile-based health technology for smoking cessation: mobile anxiety sensitivity program. Br J Clin Psychol.

[8] Businelle MS, Garey L, Gallagher MW, Hébert ET, Vujanovic A, Alexander A, Kezbers K, Matoska C, Robison J, Montgomery A, Zvolensky MJ (2022). An integrated mHealth app for smoking cessation in Black smokers with anxiety: protocol for a randomized controlled trial. JMIR Res Protoc.

[9] Businelle MS, Ma P, Kendzor DE, Frank SG, Vidrine DJ, Wetter DW (2016). An ecological momentary intervention for smoking cessation: evaluation of feasibility and effectiveness. J Med Internet Res.

[10] Spohr SA, Nandy R, Gandhiraj D, Vemulapalli A, Anne S, Walters ST (2015). Efficacy of SMS text message interventions for smoking cessation: a meta-analysis. J Subst Abuse Treat.

[11] Palmer M, Sutherland J, Barnard S, Wynne A, Rezel E, Doel A, Grigsby-Duffy L, Edwards S, Russell S, Hotopf E, Perel P, Free C (2018). The effectiveness of smoking cessation, physical activity/diet and alcohol reduction interventions delivered by mobile phones for the prevention of non-communicable diseases: a systematic review of randomised controlled trials. PLoS One.

[12] Bricker JB, Mull KE, Santiago-Torres M, Miao Z, Perski O, Di C (2022). Smoking cessation smartphone app use over time: predicting 12-month cessation outcomes in a 2-arm randomized trial. J Med Internet Res.

[13] Ono M, Schneider S, Junghaenel DU, Stone AA (2019). What affects the completion of ecological momentary assessments in chronic pain research? an individual patient data meta-analysis. J Med Internet Res.

[14] Rintala A, Wampers M, Myin-Germeys I, Viechtbauer W (2019). Response compliance and predictors thereof in studies using the experience sampling method. Psychol Assess.

[15] Ottenstein C, Werner L (2022). Compliance in ambulatory assessment studies: investigating study andsample characteristics as predictors. Assessment.

[16] Seidman AJ, George CJ, Kovacs M (2022). Ecological momentary assessment of affect in depression-prone and control samples: survey compliance and affective yield. J Affect Disord.

[17] Comulada WS, Swendeman D, Koussa MK, Mindry D, Medich M, Estrin D, Mercer N, Ramanathan N (2018). Adherence to self-monitoring healthy lifestyle behaviours through mobile phone-based ecological momentary assessments and photographic food records over 6 months in mostly ethnic minority mothers. Public Health Nutr.

[18] Jones A, Remmerswaal D, Verveer I, Robinson E, Franken IHA, Wen CKF, Field M (2019). Compliance with ecological momentary assessment protocols in substance users: a meta-analysis. Addiction.

[19] Vachon H, Viechtbauer W, Rintala A, Myin-Germeys I (2019). Compliance and retention with the experience sampling method over the continuum of severe mental disorders: meta-analysis and recommendations. J Med Internet Res.

[20] Wrzus C, Neubauer AB (2023). Ecological momentary assessment: a meta-analysis on designs, samples, and compliance across research fields. Assessment.

[21] Soyster PD, Bosley HG, Reeves JW, Altman AD, Fisher AJ (2019). Evidence for the feasibility of person-specific ecological momentary assessment across diverse populations and study designs. J Pers Oriented Res.

[22] Schüz N, Walters JAE, Frandsen M, Bower J, Ferguson SG (2014). Compliance with an EMA monitoring protocol and its relationship with participant and smoking characteristics. Nicotine Tob Res.

[23] Turner CM, Coffin P, Santos D, Huffaker S, Matheson T, Euren J, DeMartini A, Rowe C, Batki S, Santos GM (2017). Race/ethnicity, education, and age are associated with engagement in ecological momentary assessment text messaging among substance-using MSM in San Francisco. J Subst Abuse Treat.

[24] Courvoisier DS, Eid M, Lischetzke T (2012). Compliance to a cell phone-based ecological momentary assessment study: the effect of time and personality characteristics. Psychol Assess.

[25] Turner CM, Arayasirikul S, Trujillo D, Lê V, Wilson EC (2019). Social inequity and structural barriers to completion of ecological momentary assessments for young men who have sex with men and trans women living with HIV in San Francisco. JMIR Mhealth Uhealth.

[26] Wu YH, Stangl E, Oleson J, Caraher K, Dunn C (2022). Personal characteristics associated with ecological momentary assessment compliance in adult cochlear implant candidates and users. J Am Acad Audiol.

[27] Arozullah AM, Yarnold PR, Bennett CL, Soltysik RC, Wolf MS, Ferreira RM, Lee SYD, Costello S, Shakir A, Denwood C, Bryant FB, Davis T (2007). Development and validation of a short-form, rapid estimate of adult literacy in medicine. Med Care.

[28] Idler EL, Benyamini Y (1997). Self-rated health and mortality: a review of twenty-seven community studies. J Health Soc Behav.

[29] Rural-urban commuting area codes. United States Department of Agriculture.

[30] Sun J, Rhemtulla M, Vazire S (2021). Eavesdropping on missing data: what are university students doing when they miss experience sampling reports?. Pers Soc Psychol Bull.

[31] Howard AL, Lamb M (2024). Compliance trends in a 14-week ecological momentary assessment study of undergraduate alcohol drinkers. Assessment.

[32] Head KJ, Noar SM, Iannarino NT, Grant Harrington N (2013). Efficacy of text messaging-based interventions for health promotion: a meta-analysis. Soc Sci Med.

