# Correction: Smartphone-Based Survey and Message Compliance in Adults Initially Unready to Quit Smoking: Secondary Analysis of a Randomized Controlled Trial

**DOI:** 10.2196/66599

**Published:** 2024-10-15

**Authors:** Clayton Ulm, Sixia Chen, Brianna Fleshman, Lizbeth Benson, Darla E Kendzor, Summer Frank-Pearce, Jordan M Neil, Damon Vidrine, Irene De La Torre, Michael S Businelle

**Affiliations:** 1 Department of Health Behavior Gillings School of Global Public Health University of North Carolina at Chapel Hill Chapel Hill, NC United States; 2 TSET Health Promotion Research Center Stephenson Cancer Center University of Oklahoma Health Sciences Center Oklahoma City, OK United States; 3 Department of Biostatistics and Epidemiology Hudson College of Public Health The University of Oklahoma Health Sciences Center Oklahoma City, OK United States; 4 Department of Family and Preventive Medicine University of Oklahoma Health Sciences Center Oklahoma City, OK United States; 5 Department of Health Outcomes and Behavior Moffitt Cancer Center Tampa, FL United States

In “Smartphone-Based Survey and Message Compliance in Adults Initially Unready to Quit Smoking: Secondary Analysis of a Randomized Controlled Trial” (JMIR Form Res 2024;8:e56003) the authors noted an error in the authorship list.

The author Irene De La Torre was mistakenly excluded from the authorship list. Her degree is “BS” and she is affiliated to affiliation 2 of the original manuscript.

Irene De La Torre will be listed as an author immediately following Damon Vidrine and preceding Michael S Businelle.

The corrected authorship list reads as:

Clayton Ulm, Sixia Chen, Brianna Fleshman, Lizbeth Benson, Darla E Kendzor, Summer Frank-Pearce, Jordan M Neil, Damon Vidrine, Irene De La Torre, Michael S Businelle

The corresponding author has also been changed from Clayton Ulm to Michael S Businelle. In the revised manuscript, the detials of the corresponding author are listed as follows:

Michael S Businelle, PhD

TSET Health Promotion Research Center

Stephenson Cancer Center

University of Oklahoma Health Sciences Center

655 Research Parkway

Suite 400

Oklahoma City, OK, 73104

United States

Phone: 1 405 271 8001

Email: michael-businelle@ouhsc.edu

The correction will appear in the online version of the paper on the JMIR Publications website on October 15, 2024, together with the publication of this correction notice. Because this was made after submission to PubMed, PubMed Central, and other full-text repositories, the corrected article has also been resubmitted to those repositories.

