# Supplementary material for: Smartphone-Based Survey and Message Compliance in Adults Initially Unready to Quit Smoking: Secondary Analysis of a Randomized Controlled Trial
Source: JMIR Form Res. 2024 Jun 7;8:e56003. doi: 10.2196/56003 (PMC11193076; doi:10.2196/56003)
Supplement: Multimedia Appendix 1 [file formative_v8i1e56003_app1.docx]

| Table S1. Example messages. |
| --- |
| Phoenix Motivation Phase |
| 1. Pack-a-day smokers spend at least $55,000 on cigarettes over 30 years. How could you better use that money? |
| 1. After quitting smoking, ex-smokers report lower levels of stress than when they were smokers. |
| 1. Smokers often list lack of access to smoking cessation resources as a barrier to quitting. Click the "Set New Quit Date" button in the app to get access to free smoking cessation resources. |
| 1. Children, pets, and other adults can get sick by breathing in cigarette smoke. |
| Phoenix Pre-Cessation Phase |
| 1. After you quit smoking, you will be tempted to relapse. Reduce temptation by removing ashtrays, lighters, matches, and cigarette butts. These items can trigger cravings and reduce motivation for staying quit. |
| 1. During the first week or two of your quit attempt avoid drinking alcohol, or only drink in moderation when cigarettes are not available. Remember that almost 25% of all people who relapse do so while drinking. |
| 1. Try to keep busy on your quit date (e.g., visit friends/family who don't smoke, go to the movies, exercise). These activities will help you to keep your mind off smoking. |
| 1. We have found that smokers tend to underestimate the role their environment plays in increasing the likelihood of relapse. If you make it so that it takes you more than 10 minutes to get your hands on cigarettes, you may allow yourself enough time to ride out cravings and avoid relapse. |
| Phoenix Cessation Phase |
| 1. When you quit smoking, you may feel more stressed, anxious, or irritated. Try this relaxation exercise: Take a deep breath (make sure you belly goes out when you breathe in). Hold your breath and count to 10. Breathe out slowly counting to 10. Repeat 5 times. (Stress) |
| 1. Remember that the urge WILL pass! Even though it might seem like smoking would be pleasant right now, smoking will only make you feel worse later. (Urge) |
| 1. Sometimes people save a pack of cigarettes (or even a few cigarettes) “just in case” or to prove they have the willpower to not smoke. DON’T! This just makes it easier to start smoking again! (Cigarette Availability) |
| 1. Stay strong! Every day that you stay smoke free is one day closer to your new smoke-free life. (Motivation) |
| Phoenix Maintenance Phase |
| 1. Even after you are smoke free for a while, you may still feel an urge to smoke. The important thing to remember is that having an urge to smoke does not mean you have to smoke! |
| 1. If you are feeling down or low, and miss smoking, replace it with another pleasurable activity – visit a friend, or treat yourself to a good meal. |
| 1. Don’t let your guard down when you’re drinking. If a situation is tempting, leave for a few minutes until the urge passes. |
| 1. In your first days or weeks without cigarettes, you may feel down or anxious. These feelings will pass. Remind yourself that smoking is just a temporary solution. It doesn’t really fix anything. |
| Factoid Messages |
| 1. The longest living person was a French woman who lived to be 122. |
| 1. The fastest animal on earth is the Peregrine Falcon. This bird has been clocked at 200 mph, more than 3 times faster than a Cheetah. |
| 1. Heart attacks are more likely to occur on Mondays than any other day of the week. |
| 1. The speed of light (670,616,629 mph) is much faster than the speed of sound (767 mph). That is why we see lighting before we hear thunder. |
